# 04. Immunogenicity and Safety of a Quadrivalent Meningococcal Conjugate Vaccine (MenACYW-TT) Administered in Meningococcal Vaccine-Naive Participants Across a Broad Age Range (2-55 Years) in Japan

**DOI:** 10.1093/ofid/ofab466.207

**Published:** 2021-12-04

**Authors:** Osamu Matsuoka, Mugen Ujiie, Hitoshi Kikuchi, Danaya Chansinghakul, Takahiro Inoue, Kucku Varghese, Nuchra Sirisuphmitr, Tomoyuki Hashiguchi, Betzana Zambrano, Takahiro Nakama, Carina Frago, Emilia Jordanov, Mandeep S Dhingra

**Affiliations:** 1 ToCROM Clinic, Tokyo, Tokyo, Japan; 2 Center Hosp. of the Nat’l Ctr. for Global Health & Medicine, Tokyo, Tokyo, Japan; 3 Meitetsu Hospital, Aichi, Aichi, Japan; 4 Sanofi Pasteur, Bangkok, Nonthaburi, Thailand; 5 Sanofi K.K., Tokyo, Tokyo, Japan

## Abstract

**Background:**

MenACYW-TT [MenQuadfi^®^] is a quadrivalent meningococcal conjugate vaccine, licensed for use in ages 2 years and older in USA. The vaccine is also licensed in ages 12 months and older in EU and certain other countries. We evaluated the safety and immunogenicity of MenACYW-TT compared to a licensed quadrivalent conjugate meningococcal vaccine (MenACWY-DT [Menactra^®^]) in Japanese children, adolescents and adults (2-55 years of age).

**Methods:**

A phase III modified double-blind, randomized study (NCT04368429) to evaluate the immunogenicity and safety of a single dose of MenACYW-TT versus MenACWY-DT was conducted in 360 participants (ratio 1:1) between ages 2 and 55 years in Japan. Serum bactericidal assays with human complement (hSBA) were used to measure antibodies against vaccine serogroups at baseline (Day 0) and 30 days post-vaccination (D30). Safety data were collected up to 30 days post-vaccination.

**Results:**

Non-inferiority of immune responses for all four serogroups, based on percentages of participants achieving hSBA vaccine seroresponse as primary endpoint, was demonstrated for MenACYW-TT compared to MenACWY-DT at Day 30 in comparison to baseline: 85.6% vs 65.4% for serogroup A, 96.6% vs 62.6% for serogroup C, 87.4% vs 49.2% for serogroup W, and 97.7% vs 63.5% for serogroup Y. The proportions of individuals with hSBA titers ≥ 1:8 following MenACYW-TT administration were higher than those after MenACWY-DT administration for serogroups C (98.9% vs 81.0%), W (99.4% vs 91.1%) and Y (100 % vs 89.4%) and comparable for serogroup A (96.6% vs 92.7%). The hSBA GMTs were higher following administration of MenACYW-TT for all four serogroups. Immunogenicity results in participants 10 to 17 years of age and ≥ 18 years of age were comparable to those in the whole population (2-55 years). The safety profiles of MenACYW-TT and MenACWY-DT were comparable. There were no immediate adverse events (AEs), no AEs leading to study discontinuation, and no vaccine-related serious adverse events reported in the study.

**Conclusion:**

MenACYW-TT vaccine was well tolerated and demonstrated a non-inferior immune response compared to that for the licensed MenACWY-DT vaccine when administered as a single dose to meningococcal vaccine-naïve children, adolescents, and adults in Japan.

**Disclosures:**

**Osamu Matsuoka, MD**, **Sanofi Pasteur** (Scientific Research Study Investigator, Research Grant or Support) **Mugen Ujiie, MD**, **Sanofi Pasteur** (Scientific Research Study Investigator, Research Grant or Support) **Hitoshi Kikuchi, MD**, **Sanofi Pasteur** (Scientific Research Study Investigator)**Sanofi Pasteur** (Research Grant or Support) **Danaya Chansinghakul, MD**, **Sanofi Pasteur** (Employee) **Takahiro Inoue, MSc**, **Sanofi Pasteur** (Employee) **Kucku Varghese, PhD**, **Sanofi Pasteur** (Employee) **Nuchra Sirisuphmitr, MSc**, **Sanofi Pasteur** (Employee) **Tomoyuki Hashiguchi, MSc**, **Sanofi Pasteur** (Employee) **Betzana Zambrano, MD**, **Sanofi Pasteur** (Employee) **Takahiro Nakama, MD**, **Sanofi Pasteur** (Employee) **Carina Frago, MD**, **Sanofi Pasteur** (Employee, Shareholder) **Emilia Jordanov, MD**, **Sanofi Pasteur** (Employee, Shareholder) **Mandeep S. Dhingra, MD**, **Sanofi Pasteur** (Employee, Shareholder)

